# Mineral Analysis, *In Vitro* Evaluation of Alpha-Amylase, Alpha-Glucosidase, and Beta-Galactosidase Inhibition, and Antibacterial Activities of *Juglans regia* L. Bark Extracts

**DOI:** 10.1155/2021/1585692

**Published:** 2021-08-25

**Authors:** Mohamed Boulfia, Fatima Lamchouri, Hamid Toufik

**Affiliations:** Laboratory of Natural Substances, Pharmacology, Environment, Modeling, Health & Quality of Life (SNAMOPEQ), Polydisciplinary Faculty of Taza, Sidi Mohamed Ben Abdellah University of Fez, B.P. 1223, Taza-Gare, Taza, Morocco

## Abstract

In traditional medicine, various parts of the plant *Juglans regia* L. are used to treat several pathological conditions including diabetes and infectious and periodontal diseases. This includes the bark of *Juglans regia*. The present study is aimed at evaluating for the first time the mineral composition, investigating the antidiabetic and antibacterial properties of Moroccan *J. regia* bark, and finally determining the correlations between the chemical composition of the tested extracts and their biological activities. The mineral composition was determined using inductively coupled plasma atomic emission spectroscopy. Then, nine extracts were prepared by different methods and modalities of extractions and investigated for their antidiabetic activities, via tests of inhibition of alpha-amylase, alpha-glucosidase, and beta-galactosidase enzymes, and for their antibacterial activities against six strains involved in infectious diseases and periodontology. Finally, the correlation between the chemical compositions of the different extracts prepared and their antidiabetic and antibacterial potencies was determined by Principal Component Analysis (PCA). *J. regia* is an important source of mineral elements, mainly Fe (19849.8), K (3487.8), Mg (2631.03), and P (691.02) mg/kg plant material. All the extracts of *J. regia* possess antidiabetic activity, and in particular, the macerated acetone extract gave the highest inhibitory activity against alpha-amylase (IC_50_ value of 5445.33 ± 82.58 *μ*g/mL), alpha-glucosidase (IC_50_ value of 323.7 ± 1.71 *μ*g/mL), and beta-galactosidase (IC_50_ value of 811.2 ± 8.32 *μ*g/mL). For the results of antibacterial activity, the macerated acetone extract at the concentration of 80 mg/mL was found to be the most active by inducing inhibition diameters of 12, 17, 18, 11, 14.5, and 16 mm against *Escherichia coli*, *Proteus mirabilis*, *Pseudomonas aeruginosa*, *Staphylococcus aureus*, *Bacillus subtilis*, and *Listeria innocua*, respectively. PCA allowed us to deduce that the extracts richer in polyphenols, in particular, the two acetone and ethanol macerates, have a better antidiabetic activity against alpha-glucosidase as well as a better antibacterial activity. The results of the present study revealed that the aqueous and organic macerate extracts showed a better antidiabetic activity and justified the use of *J. regia* bark as an antibacterial and antiseptic agent in traditional Moroccan medicine in the treatment of dental affections.

## 1. Introduction

Diabetes mellitus is characterized by chronic hyperglycemia and disorders of carbohydrate, lipid, and protein metabolism associated with absolute or relative deficits in insulin action and/or secretion [1, 2]. The World Health Organization (WHO) has predicted that between 2014 and 2045, the number of people with diabetes will increase from 422 million to approximately 629 million [3]. Diabetes (mainly type 2 diabetes) is seeing its prevalence explode in developing countries. In Morocco, between 2011 and 2015, the number of diabetics increased from 1.5 million people to more than 2 million, an increase of 25% in 5 years. 80% of diabetes cases are type 2 and mainly are people over 40 years of age and obese [4]. However, recent studies have shown a significant increase in the number of children and adolescents with this disease, which may be associated with bad eating habits and a lack of physical activity. This leads to increasing rates of obesity, which is considered a risk condition for the development of type 2 diabetes [5].

Faced with this situation, the population has sought alternative and less expensive treatments for the diabetes disease, including the use of plants as a remedy for this disease, because the medicinal plant can have considerable hypoglycemic properties [5]. The literature analysis showed that several studies reported the benefits of different herbal treatments on several metabolic changes caused by type 2 diabetes. These studies also show the positive effects of plant extracts on normalizing body weight and decrease glucose levels, total cholesterol, and triglycerides [6–8].

In addition to diabetes, infectious diseases are a public health problem because of their frequency and severity. However, periodontal disease is defined as a bacterial infection that affects and destroys the supporting tissues of the teeth (gingiva, periodontal ligament, cementum, and alveolar bone) through an accumulation of bacterial biofilm on the surface of the teeth. This biofilm is defined as a complex microbial community of oral bacteria organized in supra- and subgingival biofilm [9]. Therefore, treatment of this biofilm with antimicrobial agents may be beneficial and necessary. Antiseptic agents, such as chlorhexidine, cetylpirinidium chloride, and fluorides, in different formulations (mouthwashes, gels), are often and still used in periodontology to potentiate the eradication of periodontal pathogens [10, 11]. However, the majority of these agents have limited subgingival efficacy, reaching only partially the subgingival biofilm and inaccessible inflamed tissue. Also, the appearance of adverse effects (dental staining, taste alteration, etc.) and bacterial resistance have often been described following prolonged use of these chemical agents [12]. Faced with these many obstacles in the use of available antibacterials, it is essential to search for new effective antibacterial substances with a broad spectrum of action. One of the strategies for this research is to explore plants used in traditional medicine.

Eastern Morocco, and more particularly the region of Taza, is characterized by a significant phytodiversity, of which several plants are used in both the treatment of diabetes and dental diseases. Among these plants, *Juglans regia* is one of the most commonly used in traditional medicine for the treatment of diabetes [13, 14]. Previous studies in Iran have even reached the stage of clinical trials by formulating 100 mg capsules of *Juglans regia* leaf extract in the treatment of diabetic patients [15–17]. Other studies have shown that the leaves have antidiarrheal, anthelmintic, and antiseptic properties and are used in the treatment of skin inflammation, ulcers [18], and cancer [19–21]. Orhan et al. reported the neuroprotective effect of extracts prepared from the leaves and fruits of *Juglans regia* harvested in Turkey [22]. Boulfia et al. have reported the antioxidant activity of aqueous and organic Moroccan *Juglans regia* bark extracts [23]. In addition, the bark of *Juglans regia* is reputed to have antibacterial and antiseptic activities, acting against bad breath and bleeding gums according to an ethnopharmacological survey that we conducted in the region of Taza, Morocco [24]. Phytochemical analysis of *Juglans regia* has been the subject of different research studies. NirmlaDevi et al. reported the presence of tannins, saponins, phenols, alkaloids, steroids, and reducing sugars in the bark of *Juglans regia* harvested in India [25]. Bou Abdallah et al. reported the presence of tocopherols, sterols, triterpenes, aliphatic alcohols, carotenoids, and volatile compounds in *Juglans regia* leaf oils in Tunisia [26]. Similarly, *Juglans regia* is an important source of steroids [22], flavonoid C-glycoside [27], flavons [22], essential oils [26, 28], tannins [29], and sesquiterpene [30].

The present study represents the continuation of our experimental studies on the Moroccan *Juglans regia* L. bark, the first part of which was devoted to the study of the effects of the use of different extraction solvents with different polarities, aqueous and organic extraction methods, and hot and cold extraction modalities by maceration on the presence and content of secondary metabolites and the antioxidant activity [23].

In the present work, we were interested in determining for the first time the mineral composition of the Moroccan *Juglans regia* L. bark and in evaluating the antidiabetic activity via three tests of inhibition of the enzymes alpha-amylase, alpha-glucosidase, and beta-galactosidase of its aqueous and organic extracts (ethanolic, acetonic, and diethyl ether) prepared hot by Soxhlet extraction and cold by maceration. Subsequently, we were also interested in studying their antibacterial activity by the method of diffusion on disc and in determining the minimum inhibitory and bactericidal concentrations of the extracts which proved to be active. Finally, we used Principal Component Analysis (PCA) to reveal the correlations between the chemical composition of the tested extracts and the results of the study of its antidiabetic and antibacterial activities.

## 2. Materials and Methods

### 2.1. Chemicals and Reagents

Alpha-amylase from *Aspergillus oryzae*, starch, sodium phosphate buffer, dinitrosalicylic acid (DNS), acarbose, 4-p-nitrophenyl-*α*-D-glucopyranoside (pNPG), sodium carbonate (Na_2_CO_3_), alpha-glucosidase from *Saccharomyces cerevisiae*, beta-galactosidase from *Escherichia coli*, 2-nitrophenyl *β*-D-galactopyranoside, quercetin, Müeller-Hinton's agar, (3-(4,5-dimethyl-2-thiazolyl)-2,5-diphenyl-2H-tetrazolium bromide), nitric acid, perchloric acid, diethyl ether, acetone, ethanol, and dimethyl sulfoxide chemicals were purchased from Sigma Aldrich (Saint-Louis, Missouri, USA).

### 2.2. Plant Material

*Juglans regia* material was collected in November 2019 in the commune of Oued Amlil (geographical coordinates: 34°16.604′N 004°01.709′W; altitude: 497 m), Province of Taza, Morocco, and the plant was identified by Dr. Abdelmajid Khabbach in the Laboratory of Natural Substances, Pharmacology, Environment, Modeling, Health & Quality of Life (SNAMOPEQ), Polydisciplinary Faculty of Taza (FPT), Sidi Mohamed Ben Abdellah University of Fez, Morocco. A voucher specimen was deposited in the herbarium under the code SA 2016/08. The bark of *Juglans regia* was thoroughly cleaned of unwanted material, cut into small pieces, and dried in the shade at room temperature until constant weight and was then powdered and used for further investigation.

### 2.3. Mineral Content

According to the method of Arora et al. [31], the mineral composition of *Juglans regia* bark was investigated. Mineral elements (potassium (K), calcium (Ca), magnesium (Mg), sodium (Na), phosphorus (P), copper (Cu), iron (Fe), selenium (Se), strontium (Sr), and zinc (Zn)) were determined using inductively coupled plasma atomic emission spectroscopy (ICP-AES) (HORIBA Jobin Yvon). Thus, 0.1 mg of *J. regia* bark was digested with nitric acid and perchloric acid (25% : 75%) solution, before being incinerated at 110°C, then brought back dry until the mineralization was discolored on a sand bath. The residue was dissolved in 10 mL HCL (5%), and the contents were filtered through 0.45 *μ*m porosity filters until a clear solution was obtained. The sample solution was made up to a final volume of 25 mL with distilled water and analyzed by atomic absorption spectrophotometry (ICP-AES) (HORIBA Jobin Yvon).

### 2.4. Preparation of Plant Extracts

*Juglans regia* bark (100 g) was extracted using separately four increasing polarity solvents (diethyl ether, acetone, ethanol, and water) by different methods and modalities as described in our previous work [23]. Nine extracts were obtained, namely, three aqueous lyophilized extracts (decocted, infused, and macerated) and six organic extracts (ethanolic, macerated ethanolic, acetone, macerated acetone, diethyl ether, and macerated diethyl ether) [23].

### 2.5. Qualitative and Quantitative Phytochemical Analysis

To determine the chemical composition of *J. regia* bark, two analyses have been conducted; the first one was aimed at investigating the presence or absence of the main secondary metabolites (alkaloids, tannins, saponins, anthraquinones, flavonoids, quinones, sterols, and anthracenosides) by staining and/or precipitation reactions, and the second one sought to quantify the content of polyphenols, flavonoids, and tannins present in aqueous and organic extracts of *J. regia* bark [23].

### 2.6. Evaluation of the Antidiabetic Effect

#### 2.6.1. Alpha-Amylase Inhibitory Activity

The alpha-amylase inhibitory potentials were investigated by reacting different concentrations of the extracts with alpha-amylase enzyme and starch solution, according to the method of Wickramaratne et al. [32]. A mixture of 200 *μ*L of samples and 200 *μ*L of 0.02 M sodium phosphate buffer (pH = 6.9) containing the enzyme alpha-amylase (2 U/mL) was incubated at 30°C for 10 min. Then, 200 *μ*L of 1% starch solution in 0.02 M sodium phosphate buffer (pH = 6.9) was added to the reacting mixture. Therefore, the reaction mixture was incubated at 30°C for 3 min. Thereafter, 200 *μ*L of dinitrosalicylic acid (DNS) was added, and the reaction mixture was incubated in a boiling water bath at 85-90°C for 10 min. Then, the reaction mixture was diluted by adding 5 mL of distilled water, and absorbance was measured at 540 nm in the spectrophotometer (SPECUVIS2 UV/Vis, No: HF1309003). Acarbose was used as a positive control.

The alpha-amylase inhibitory activity was expressed as percent inhibition and was calculated as follows:

(1) $$\begin{array}{c} \text{Inhibition }\left(\%\right)=100\times \left[\frac{\left(\text{A}1–\text{A}2\right)-\left(\text{A}3–\text{A}4\right)}{\left(\text{A}1–\text{A}2\right)}\right], \end{array}$$

where A1 refers to the absorbance of the control (enzyme + buffer), A2 refers to the absorbance of control blank (buffer without enzyme), A3 refers to the absorbance of the sample (enzyme + extract), and A4 is the absorbance of sample blank (extract without enzyme).

#### 2.6.2. Alpha-Glucosidase Inhibitory Activity

The inhibitory potency of *J. regia* extracts against alpha-glucosidase was evaluated by measuring the formation of 4-nitrophenol by alpha-glucosidase after reaction with 4-p-nitrophenyl-*α*-D-glucopyranoside (pNPG) according to the method of Lordan et al. [33]. Thus, the reaction mixture containing 150 *μ*L of extracts at various concentrations and 100 *μ*L of alpha-glucosidase solution (0.1 U/mL) was preincubated at 37°C for 10 min. Subsequently, 200 *μ*L of 1 mM pNPG solution in sodium phosphate buffer (0.1 M/pH = 6.7) was added and incubated at 37°C for 30 min. The reaction was terminated by adding 1 mL of sodium carbonate solution (Na_2_CO_3_/0.1 M), and the absorbance was measured at 405 nm. Acarbose was included as a positive control. The percent inhibition of alpha-glucosidase was determined as described in the alpha-amylase assay, and the IC_50_ values were determined.

#### 2.6.3. Beta-Galactosidase Inhibitory Activity

The beta-galactosidase inhibition activity was evaluated using the method of Maruhn in 1976 [34]. For the inhibition of the beta-galactosidase enzyme, a mixture of 150 *μ*L of various extract concentrations and 100 *μ*L of sodium phosphate buffer (0.1 M/pH = 7.6) containing the solution of enzyme beta-galactosidase (1 U/mL) was incubated at 37°C for 10 min. Then, we added 200 *μ*L of 2-nitrophenyl *β*-D-galactopyranoside (1 mM) solubilized in sodium phosphate buffer (0.1 M/pH = 7.6). Reaction mixtures were incubated at 37°C for 30 min. After incubation, 1 mL of Na_2_CO_3_ was added to stop the reaction, and absorbance was measured at 410 nm. Quercetin was used as a positive control. The percent inhibition of beta-galactosidase was determined as described in alpha-amylase and alpha-glucosidase assays. The concentration of the sample that inhibits the enzyme by 50% was evaluated.

### 2.7. Evaluation of the Antibacterial Effect

#### 2.7.1. Bacterial Strains

To determine the antibacterial activity of *J. regia* extracts, 6 bacterial strains involved in periodontal and infectious diseases were used: 3 Gram-positive bacteria—*Staphylococcus aureus* (CECT976), *Bacillus subtilis* (DSM6633), and *Listeria innocua* (CECT 4030), and 3 Gram-negative bacteria—*Escherichia coli* (K12), *Proteus mirabilis*, and *Pseudomonas aeruginosa* (CECT118).

#### 2.7.2. Preparation of Bacterial Suspensions

To prepare the bacterial suspensions, three to four well-isolated colonies were selected and emulsified in a tube containing sterile saline solution to have turbidity near that of McFarland 0.5 standards; the suspensions were then diluted in tubes containing sterile saline solution to have a final concentration of 10^8^ CFU/mL. An inoculum of 100 *μ*L of each bacterial suspension was plotted in petri dishes, each containing 20 mL of Müeller-Hinton's agar, and left hanging for 5 min before inserting the discs.

#### 2.7.3. Disc Diffusion Method

The experimental protocol consisted of depositing a sterile Whatman paper disc (6 mm in diameter) impregnated with different concentrations of the prepared extracts of *J. regia* bark (20, 40, and 80 mg/mL) dissolved in 10% of DMSO. The dishes were left for 15 min at room temperature and then incubated at 37°C for 20 h. After incubation, the antibacterial effect was evaluated by measuring the inhibition zones in millimeters against the tested bacterial strains [35]. The experiments were repeated three times. Negative controls were performed by DMSO at 10%, while tetracycline and amikacin were used as positive controls.

#### 2.7.4. Determination of Minimum Inhibitory Concentration (MIC)

This technique consists of making successive dilutions in a liquid culture medium (Müeller-Hinton) in microplates of 96 sterile wells, from a stock solution of the extracts to be tested and whose concentration is known (80 mg/mL). To these dilutions, a microorganism suspension (10 *μ*L) with a concentration of 10^8^ CFU/mL was added to each well. Afterward, the plates were incubated at 37°C for 24 hours. Then, 10 *μ*L of MTT solution (3-(4,5-dimethyl-2-thiazolyl)-2,5-diphenyl-2H-tetrazolium bromide) was added to each well, and the plate was reincubated for 15 minutes at 37°C. The results were read visually, and the appearance of a purple stain shows bacterial growth. The lowest concentration which had no visible growth after 24 h incubation was considered as the MIC [36].

#### 2.7.5. Determination of Minimum Bactericidal Concentration (MBC)

To determine the MBC values, well solutions with an extract concentration equal to or higher than the MIC values were used. 10 *μ*L from each well was subcultured on nutrient agar (Müeller-Hinton's agar) in petri dishes. The bacterial cultures were incubated at 37°C for 24 h. The minimum concentration that had no visible growth on agar plates after 24 h incubation was considered as the MBC.

### 2.8. Statistical Analysis

The results were expressed as the mean ± standard error. Nonlinear regression analysis was adopted to determine the IC_50_ values of the three assays (alpha-amylase, alpha-glucosidase, and beta-galactosidase assays). The data were analyzed by one-way analysis of variance (one-way ANOVA), Turkey, and the procedure consisted of comparing all pairs of columns for the significance of difference. A difference in the mean values of *p* < 0.05 was considered to be statistically significant. Analysis was performed with GraphPad Prism® 5.0 software. Principal Component Analysis (PCA) was performed by the XLSTAT software.

## 3. Results

### 3.1. Mineral Composition of *Juglans regia* L. Bark

According to our bibliographic research, the present study represents the first international study dedicated to the evaluation of the mineral composition of *J. regia* bark. The results of this study are expressed in milligrams per kilogram of plant material (mg/kg) and are represented in Table 1. This shows that *J. regia* bark is an important source of mineral elements, with very high contents of Fe (19849.8), K (3487.8), Mg (2631.03), P (691.02), Na (515.75), Ca (256.98), Cu (189.75), and Sr (18.18) mg/kg of plant material. For both elements Se and Zn, we recorded lower values (<0.01 mg/L).

### 3.2. Evaluation of the Antidiabetic Effect of *Juglans regia* L. Bark

#### 3.2.1. Alpha-Amylase Inhibitory Activity

According to our bibliographic research, there are many studies of the antidiabetic activity of *J. regia* leaves, but few studies evaluated this activity on the bark of *J. regia.* The present study represents for the first time the results of alpha-amylase, alpha-glucosidase, and beta-galactosidase inhibition of aqueous and organic extracts of *J. regia* bark and are presented in Table 2. From this table, we noticed that for the alpha-amylase inhibition assay, aqueous extracts are less active than organic extracts. For the aqueous extracts, the extract prepared by infusion was the most active with an IC_50_ of about 8689.66 ± 318.23 *μ*g/mL, twice as active as that prepared by decoction (IC_50_ = 16161 ± 62.61 *μ*g/mL). For the organic extracts, the macerated acetone showed the best alpha-amylase inhibition activity with an IC_50_ of about 5445.33 ± 82.58 *μ*g/mL. Compared to the reference standard, acarbose exhibited an IC_50_ in the range of 616.33 ± 6.58 *μ*g/mL which remains significantly active than the aqueous and organic extracts of *J. regia* bark.

#### 3.2.2. Alpha-Glucosidase Inhibitory Activity

The results obtained from the evaluation of the alpha-glucosidase inhibition activity of the aqueous and organic extracts of *J. regia* are listed in Table 2 and show that the two extracts prepared by Soxhlet extraction and by cold maceration by the acetone solvent proved to be the most active with a nonsignificant difference (acetone extract: IC_50_ = 306.9 ± 1.88 *μ*g/mL; macerated acetone: IC_50_ = 323.7 ± 1.71 *μ*g/mL). The aqueous extract prepared by cold maceration showed an interesting hypoglycemic effect with an IC_50_ of about 414.46 ± 13.98 *μ*g/mL. All tested extracts were found to be less active than the reference standard, acarbose, which showed an IC_50_ value of 195 ± 5 *μ*g/mL which is significantly different from the aqueous and organic extracts of *J. regia* (*P* < 0.05).

#### 3.2.3. Beta-Galactosidase Inhibitory Activity

The different extracts prepared from the *J. regia* bark were tested for their hypoglycemic activity by the beta-galactosidase inhibition assay, and the results obtained are represented in Table 2. These showed that all the extracts tested have a beta-galactosidase inhibition capacity with a significant difference (*P* < 0.05) between aqueous and organic extracts and that the latter is more active than the aqueous extracts. The aqueous macerate showed an IC_50_ in the range of 960.1 ± 38.43 *μ*g/mL, while the macerated acetone has high inhibitory power with an IC_50_ of 811.2 ± 8.32 *μ*g/mL.

### 3.3. Evaluation of the Antibacterial Effect

The antibacterial activity of organic extracts prepared from *J. regia* bark was evaluated against a set of bacterial strains including both Gram-positive bacteria (*Staphylococcus aureus* (CECT976), *Bacillus subtilis* (DSM6633), and *Listeria innocua* (CECT 4030)) and Gram-negative bacteria (*Escherichia coli* (K12), *Proteus mirabilis*, and *Pseudomonas aeruginosa* (CECT118)). Antibacterial potency was evaluated by measuring the diameter of the zone of inhibition in mm and determining MIC and BMC values.

#### 3.3.1. Disc Diffusion Method

The results of antibacterial activity by disc diffusion method are reported in Table 3; we can note that the organic extracts prepared from *J. regia* bark exhibit a wide spectrum of the zone of inhibition which varies depending on the strain tested, the extract tested, and the concentration used.

For the disc diffusion method, an extract is considered active when it induces a zone of inhibition greater than or equal to 10 mm [37]. According to Table 3, all extracts were active against all tested strains. Thus, the macerated acetone extract at the concentration of 80 mg/mL was found to be the most active by inducing the inhibition diameters of 12, 17, 18, 11, 14.5, and 16 mm on *Escherichia coli*, *Proteus mirabilis*, *Pseudomonas aeruginosa*, *Staphylococcus aureus*, *Bacillus subtilis*, and *Listeria innocua*, respectively. Similarly, the macerated diethyl ether extract at the same concentration showed high antibacterial power with inhibition diameters of 14, 21.5, 10, 14.5, 20, and 11 mm on *Escherichia coli*, *Proteus mirabilis*, *Pseudomonas aeruginosa*, *Staphylococcus aureus*, *Bacillus subtilis*, and *Listeria innocua*, respectively. It should be noted that the inhibition diameters induced by all these extracts remain higher than those of the reference antibiotic, amikacin, which showed no zone of inhibition against *Escherichia coli*, *Proteus mirabilis*, and *Staphylococcus aureus* strains. Similarly, tetracycline was active only against *Escherichia coli*, *Proteus mirabilis*, and *Staphylococcus aureus* strains with inhibition diameters of 13, 23, and 13 mm, respectively.

#### 3.3.2. Minimal Inhibitory Concentration (MIC) and Minimal Bactericidal Concentration MBC)

The purpose of the microdilution method is to evaluate minimum inhibitory concentrations and determine the lowest concentration of an antibacterial agent necessary to inhibit the growth of a microorganism.

The present study allowed us to determine the minimum inhibitory concentration of the different organic extracts of *J. regia* bark and then to determine the minimum bactericidal concentration of our extracts, which allowed us to quantify and compare these results with those of the disc diffusion method, but also to characterize the nature of the effect revealed by an extract on a given microorganism.

Table 4 summarizes the results obtained from the minimum inhibitory and bactericidal concentrations of organic extracts of *J. regia.* According to this table, we notice that all the tested extracts had important antibacterial activities with MIC values between 0.87 mg/mL and 5 mg/mL, and the macerated acetone extract was the most active with MIC values of 0.87 mg/mL against *Proteus mirabilis*, *Pseudomonas aeruginosa*, and *Listeria innocua* and MIC values of 1.75 mg/mL against *Escherichia coli* and *Staphylococcus aureus*.

The MBC/MIC ratio was also calculated to highlight the nature of the antibacterial effect of the extracts tested. When this ratio is lower than 4, the extract is considered a bactericidal extract, and when it is higher than 4, it is considered a bacteriostatic extract [38].

According to Table 4, it appears that all the organic extracts tested do not have a bactericidal effect but rather a bacteriostatic power; it is the case of the macerated acetone which exerts a bacteriostatic effect against 4 strains, namely, *Escherichia coli*, *Pseudomonas aeruginosa*, *Staphylococcus aureus*, and *Listeria innocua*, followed by the ethanolic extract with a bacteriostatic effect against 3 strains, namely, *Escherichia coli*, *Pseudomonas aeruginosa*, and *Listeria innocua*. The other extracts have a bacteriostatic power on one or two strains (Table 4).

### 3.4. Principal Component Analysis (PCA)

To determine the correlations between the chemical composition [23] and the different pharmacological activities evaluated in this work, namely, antidiabetic and antibacterial, we calculated the correlation matrix based on the results of the determination of polyphenols, flavonoids, and tannins and the results of the antidiabetic and antibacterial activities represented as variables, and the individuals are the nine aqueous and organic extracts of *J. regia* bark (Table 5).

From Table 5, we noticed that the richer the extract is in polyphenols, the better the correlation with the alpha-glucosidase inhibition test and the antibacterial activity represented by the *Pseudomonas aeruginosa* strain with a correlation coefficient of *r* = 0.7904 and 0.9595, respectively. The extracts that are rich in tannins (macerated diethyl ether and macerated acetone) show positive correlations with the strains of antibacterial activity of *Escherichia coli*, *Staphylococcus aureus*, and *Bacillus subtilis* with a correlation coefficient of *r* = 0.9511, *r* = 0.7969, and *r* = 0.8818, respectively. A weak correlation was obtained between alpha-amylase and alpha-glucosidase enzyme inhibition tests and flavonoid content with correlation coefficients of *r* = 0.3819 and *r* = 0.0787, respectively.

**Figure 1:**
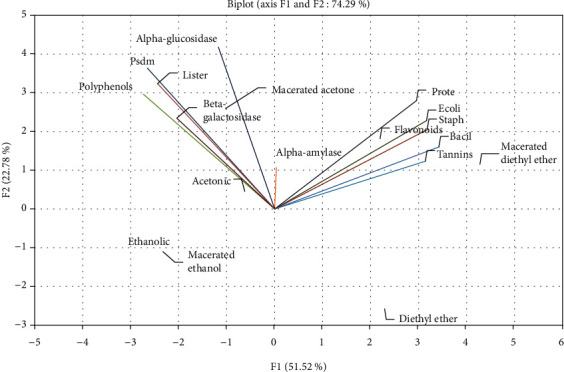
Graphical representation of the Principal Component Analysis (PCA) performed by the values of different chemical families dosed and the tests of the antidiabetic and antibacterial activities. Ecoli: *Escherichia coli* (K12). Prote: *Proteus mirabilis.* Psdm: *Pseudomonas aeruginosa* (CECT118). Staph: *Staphylococcus aureus* (CECT976). Bacil: *Bacillus subtilis* (DSM6633). Lister: *Listeria innocua* (CECT 4030).

The distribution of the tests of antidiabetic and antibacterial activity with the organic extracts of *J. regia* bark on the first two axes F1 and F2 of the PCA are presented in Figure 1. According to the latter, we notice that the two-axis F1 and F2 explain 74.29% of the retained information; the first component F1 explains 51.52% of the total information, and the second component F2 shows 22.78% of the total information.

The F1 axis is mainly constituted by the positive correlation between polyphenols, tannins, and bacterial strains *Escherichia coli*, *Proteus mirabilis*, *Pseudomonas aeruginosa*, *Staphylococcus aureus*, *Bacillus subtilis*, and *Listeria innocua*. The F2 axis is formed mainly by the alpha-glucosidase inhibition test (Figure 1).

## 4. Discussion

### 4.1. Mineral Composition of *Juglans regia* L. Bark

According to our literature search, there have been no previous studies that evaluated the mineral content of *J. regia* bark and our study is the first one that assayed the mineral elements present in this part of the plant.

The walnut (*J. regia* L.), which belongs to the *Juglandaceae* family, is one of the nuts commonly found in the Mediterranean diet. Recently, the walnut has been considered a natural functional food of great economic interest because of its nutritional and medicinal benefits. Moreover, the nut contains many beneficial compounds, such as polyunsaturated fatty acids and proteins. The beneficial effect of nut consumption against many diseases has been reported, including protection against diabetes [39]. In addition, the bark of *J. regia* is a part of the plant widely used by the population in traditional medicine, because of its anti-inflammatory, anticancer, depurative, diuretic, and laxative properties [25]. The antioxidant properties of *J. regia* bark have been studied and reported in our previous work [23]. In addition, the mineral elements of a plant can influence its medicinal properties. Therefore, the present study is aimed at quantifying the content of mineral elements present in the bark of *J. regia*.

From Table 1, we noticed that *J. regia* is an important source of both macroelements (calcium (Ca), potassium (K), magnesium (Mg), sodium (Na), and phosphorus (P)) and microelements (copper (Cu), iron (Fe), strontium (Sr)) which play an important role in human diet due to their antioxidant power. Indeed, calcium is the most abundant mineral in the body, with 1000 to 1500 g found in adults; it is involved in many reactions in our cells at the enzyme level. Phosphorus plays a role in the constitution of cells. Sodium is the predominant element in the blood and the extracellular fluids of the body; it determines the water balance of the organism and the hydration of the cells (with potassium) and plays a role in maintaining the acid-base balance. Magnesium has an important physiological role; it stimulates the formation of antibodies, necessary in many enzyme systems, especially those related to energy production [40]. Our results are in agreement with the results of the mineralogical analysis of the walnut conducted by Tapia et al. which revealed values of Ca of 133 mg/100 gr of fresh plant material, K of 370 mg/100 gr, Cu of 1.47 mg/100 gr, Na of 4.8 mg/100 gr, Mg of 419 mg/100 gr, Mn of 2.2 mg/100 gr, Fe of 2.1 mg/100 gr, and Zn of 1.67 mg/100 gr of the fresh plant material of the Serr variety of the nut [41]. The absence of zinc in *J. regia* bark can be explained by the geographical origin and the part of the plant used.

In comparison with another plant collected in the region of Taza, named *Leopoldia comosa*, the investigations of the mineral composition shows that the bulb of *Leopoldia comosa* has very high contents of Fe and K with values, respectively, of 33552 and 1843.14 mg/kg of plant matter, followed by P (756.36 mg/kg), Na (439.65 mg/kg), Cu (303.9 mg/kg), Mg (272.37 mg/kg), and Ca (20.55 mg/kg of plant matter) [42]. The richness of the bulb of *Leopoldia comosa* by mineral elements can explain their food use in the Mediterranean countries.

### 4.2. Antidiabetic Activity of *Juglans regia* L. Bark

Due to the lack of studies evaluating the hypoglycemic properties of *J. regia* bark, the present study seeks to evaluate and document these properties and subsequently compare the activity of different parts of the same plant that would allow the use of the right part of the plant to have the best activity.

From our study, we noticed that all the extracts of *J. regia* bark showed inhibition activity of alpha-amylase, alpha-glucosidase, and beta-galactosidase enzymes and that this activity varies from aqueous to organic extracts and also from one enzyme to another, which can be explained by the use of different extraction modalities that we have adopted in our study and which consequently impacted the presence or absence of secondary metabolites with varying concentrations between aqueous and organic extracts and also between extracts prepared with Soxhlet extraction or cold by maceration. Similarly, the choice of using different tests to evaluate the antidiabetic activity influenced the results obtained by allowing to differentiate between the extracts tested according to their mechanisms of action.

For the alpha-amylase assay, the infused extract was the most active of the aqueous extracts with an IC_50_ = 8689.66 ± 318.23 *μ*g/mL, and for the organic extracts, the macerated acetone exhibited the greatest inhibitory power with an IC_50_ = 5445.33 ± 82.58 *μ*g/mL.

Of the nine extracts tested, the two aqueous and acetone macerates were the most active via the two alpha-glucosidase enzyme inhibition tests (aqueous macerate: IC_50_ = 414.46 ± 13.98 *μ*g/mL; acetone macerate: IC_50_ = 323.7 ± 1.71 *μ*g/mL) and *β*-galactosidase whose aqueous macerate showed an IC_50_ value of 960.1 ± 38.43 *μ*g/mL while the acetone macerate has a high inhibition power with an IC_50_ value of 811.2 ± 8.32 *μ*g/mL. This difference in activity between aqueous and organic extracts is probably due to the difference in the content of flavonoids, tannins, and phenolic compounds in each extract as described previously in our previous work [23]. Indeed, the flavonoid, tannin, and polyphenol contents of the two macerates are, respectively, 48.418 ± 0.087 *μ*g QE/mg E, 0.083 ± 0.036 *μ*g CE/mg E, and 30.519 ± 0.012 *μ*g GAE/mg E for the aqueous macerate and 1277.981 ± 2.911 *μ*g QE/mg E, 38.056 ± 1.886 *μ*g CE/mg E, and 327.972 ± 0.06 *μ*g GAE/mg E for the acetone macerate [23].

In comparison with a plant from the region of Taza studied in our laboratory under the same experimental conditions, Bouabid et al. reported that the two aqueous and methanolic macerate extracts prepared from the underground part of *Atractylis gummifera* L. (*Asteraceae*) were found to have an inhibitory effect of alpha-amylase enzyme with an IC_50_, respectively, in the order of 1000 ± 0.055 *μ*g/mL and 557 ± 0.013 *μ*g/mL. Similarly, for the alpha-glucosidase test, the aqueous and methanolic macerates presented IC_50_ values of the order of 1461 ± 0.047 *μ*g/mL and 743 ± 0.017 *μ*g/mL. For the beta-galactosidase test, the aqueous macerate presented with an IC_50_ of the order of 2230 ± 0.012 *μ*g/mL, while on the other hand, the methanolic macerate presented with an IC_50_ of the order of 2443 ± 0.071 [43]. These results are better than those obtained for extracts prepared from *Juglans regia* bark (*Juglandaceae*), which can be explained by the difference of the botanical family and therefore the presence of other chemical families. The results of the hypoglycemic power of *Atractylis gummifera* extracts justify their traditional uses by the population in the treatment of diabetes [13]. Several previous works have demonstrated that phenolic compounds can influence carbohydrate metabolism at different levels, improving postprandial glycemic levels, fasting blood glucose, insulin secretion, and insulin sensitivity, being that a strategy for the prevention of type 2 diabetes is aimed at limiting the rate of blood glucose uptake through the intestines [44, 45]. In comparison with other studies performed on *J. regia*, Rahimzadeh et al. [46] reported that the aqueous macerated extract prepared from the leaves of this plant harvested in Iran has a very high alpha-amylase inhibitory power with an IC_50_ value of 320 ± 0.07 *μ*g/mL, which is still more than 40 times more active than the one we found for the aqueous macerate in our study with an IC_50_ = 13424.33 ± 284.55 *μ*g/mL. The result can be explained by the geographical variation of the harvest of the plant, the methods, and modalities of extractions used, and also the part of the plant that can influence the medicinal properties of a plant. Another study performed on the leaves of *J. regia* reported that the hydroalcoholic extract of this plant harvested in Iran at the concentration of 200 mg/kg of rat weight resulted in a significant decrease in blood glucose, glycosylated hemoglobin, LDL, triglycerides, and total cholesterol and a significant increase in insulin and HDL levels [17]. Similarly, the study by Javidanpour et al. reported that the methanolic extract of *J. regia* leaves harvested in Iran significantly decreased the level of glucose (*p* < 0.05) and glycosylated hemoglobin (*p* = 0.001) in streptozotocin-induced diabetic rats [47].

### 4.3. Antibacterial Activity: Disc Diffusion Method, Minimum Inhibitory Concentration (MIC), and Minimum Bactericidal Concentration (MBC) of Organic Extracts of *Juglans regia* L. Bark

The results obtained with Moroccan *J. regia* L. bark are similar and in some cases better than those obtained with different extracts of *J. regia* bark collected from other countries, such as Iran, Portugal, Tunisia, India, and Saudi Arabia. The study conducted by Zakavi et al. on the bark of *J. regia* harvested in Iran showed inhibition zones of 12, 15, 11, and 0 mm on *Staphylococcus aureus*, *Streptococcus sanguis*, *Streptococcus salivarius*, and *Streptococcus mutans* at the concentration of 500 mg/mL for the aqueous extract, while on the other hand, the ethanolic extract was less active with inhibition zones of 8, 8.75, and 0 mm on the strains previously described [48]. Another study carried out in Iran by Bakhtiari and Khalafi [49] on the hydroalcoholic macerated extract of *J. regia* bark showed a zone of inhibition of 16.2, 14, 14.2, and 13.3 mm, respectively, on *Staphylococcus aureus*, *Pasteurella multocida*, *Mannheimia haemolytica*, and *Streptococcus* spp. at the concentration of 250 mg/mL. These results are still higher than those of the study that was conducted by Zakavi et al. [48]. This can be explained by the difference in the extraction modality and the choice of solvent used for extraction. In comparison with our study, our results are superior to those obtained by Zakavi et al. [48]; we found a 10 mm zone of inhibition on *Staphylococcus aureus* and only at the concentration of 80 mg/mL of the ethanolic extract prepared by Soxhlet extraction. Another study by Noumi et al. [50] revealed that the three extracts prepared by cold maceration using solvents such as methanol, ethyl acetate, and acetone for 24 hours are active and present a zone of inhibition that differs depending on the microorganism used and the extract tested. The three extracts methanol, ethyl acetate, and acetone macerates have a zone of inhibition of 13.66, 19.66, and 13.33 mm, respectively, on the strain *Staphylococcus aureus* ATCC 25923, and they have a zone of inhibition of about 8, 10, and 8 mm on the *Pseudomonas aeruginosa* strain, while in our study, the acetone macerate showed a zone of inhibition of 11 mm and the diethyl ether macerate showed a zone of 14.5 mm on the *Staphylococcus aureus* strain CECT976 and a higher zone of inhibition of 18 mm for the acetone macerate on *Pseudomonas aeruginosa* compared to that obtained with Noumi et al. [50]. Sharma et al. [51] tested the antibacterial activity of ethanolic, ethyl acetate, and aqueous extracts on four strains: *Escherichia coli*, *Bacillus subtilis*, *Klebsiella aerogenosa*, and *Staphylococcus aureus*. The three extracts showed a zone of inhibition of 16 to 20 mm at the concentration of 5 mg/mL on the different strains, and the ethanolic extract showed a zone of inhibition of 16.2, 16, 16.8, and 16.4 mm on *Escherichia coli*, *Bacillus subtilis*, *Klebsiella aerogenosa*, and *Staphylococcus aureus*, respectively. These results are better than those we found and can be explained by the difference of the part of the plant used which is the green husk and the environment where the plant was harvested (India) which have consequences on the presence of the different chemical families. Ara et al. [37] tested the antibacterial activity of two samples of *J. regia* harvested from different regions, the first one harvested in Afghanistan (MHO) and the second one purchased in Saudi Arabia (MHC). The methanolic extract (MHO) prepared cold by maceration for one week showed significant activity with inhibition zones of 20, 20, 13, 28, 15, 30, and 25 mm, respectively, on *Salmonella suis* ATCC 13076, *Pseudomonas aeruginosa* ATCC 27583, *Escherichia coli* ATCC 25922, *Shigella sonnei* ATCC 11060, *Bacillus subtilis* ATCC 6633, *Staphylococcus aureus* ATCC 25923, and *Candida albicans* ATCC 10231. On the other hand, the methanolic extract (MHC) from Saudi Arabia showed a low zone of inhibition of 17, 25, 15, 28, 16, 25, and 25 mm, respectively, on the previously described strains. The method of cold extraction by maceration for a long time seems to extract phenolic compounds to which some researchers have reported their important roles in antibacterial activity [52, 53].

Another study carried out by Pereira et al. [27] on the leaves of *J. regia* from Portugal intended to test the antimicrobial effect of six walnut trees of different varieties against Gram-positive bacteria (*Bacillus cereus*, *Bacillus subtilis*, and *Staphylococcus aureus*) and Gram-negative bacteria (*Pseudomonas aeruginosa*, *Escherichia coli*, and *Klebsiella pneumoniae*) and whose results showed that Gram-positive bacteria (*B. cereus*, *B. subtilis*, and *S. aureus*) were inhibited by the aqueous extract (Decocted) of the Lara variety at very low concentrations, showing a MIC value of 0.1 mg/mL for *B. cereus* and *S. aureus* and a MIC of 10 mg/mL for *B. subtilis. Staphylococcus* aureus was also inhibited by the Mayette, Parisienne, and Marbot varieties but at a higher extract concentration (1 mg/mL), while Gram-negative bacteria (*P. aeruginosa*, *E. coli*, and *K. pneumoniae*) were less sensitive for the decocted extract of 6 walnut varieties (Lara, Franquette, Mellanaise, Mayette, Parisienne, and Marbot). Indeed, the decocted extract prepared from 6 walnut varieties inhibited bacterial growth by exhibiting a MIC of 100 mg/mL for *P. aeruginosa*, *E. coli*, and *K. pneumoniae* strains, respectively. These results agree with those found by Rather et al. [54] who reported that the essential oils of *J. regia* leaves show strong antimicrobial power, with MIC values of 15.62, 15.62, 32.25, 31.25, 15.62, 62.5, 62.5, 31.25, and 62.5 *μ*g/mL on *B. subtilis*, *S. epidermidis*, *P. vulgaris*, *P. aeruginosa*, *S. aureus*, *S. typhi*, *E. coli*, *S. dyssenteriae*, and *K. pneumonia*, respectively. In the study conducted by Zakavi et al. on *J. regia* bark harvested in Iran, the MIC values of the ethanolic extract are in the order of 2, 1.25, 2.5, and 5 mg/mL on *Staphylococcus aureus*, *Streptococcus sanguis*, *Streptococcus salivarius*, and *Streptococcus mutans* and the MIC values of the aqueous extract are in the order of 125, 2.5, and 2.5 mg/mL on *Staphylococcus aureus*, *Streptococcus sanguis*, *Streptococcus salivarius* [48].

Our results disagree with the work realized by Senhaji et al. [55] on the antibacterial activity of *Anabasis aretioïdes* extracts prepared under the same operating conditions and using the same strains, which advanced a total absence of the antibacterial activity of the petroleum ether extract whose polarity is close to that of the diethyl ether used in our study and which gave promising results. Hence, there is a necessity and the interest to have extracts prepared by solvents of different polarities and according to various extraction modalities.

The antibacterial power of all our organic extracts of *J. regia* bark is explained by the results of the phytochemical study, which revealed the presence of flavonoids, tannins, phenolic compounds, sterols, anthraquinones, quinones, and saponins [23] and whose antimicrobial properties have already been demonstrated [56–59]. Most of our extracts showed very high flavonoid, tannin, and polyphenol contents, in the first order, the acetone macerated extract whose contents are in the order of 1277.981 ± 2.911 *μ*g QE/mg E, 38.056 ± 1.886 *μ*g CE/mg E, and 327.972 ± 0.06 *μ*g GAE/mg E, respectively, for flavonoids, tannins, and polyphenols [23] which allows us to conclude that flavonoids, tannins, and polyphenols have an antibacterial action, which is in line with the literature, which reported the antimicrobial activity of polyphenols. Among polyphenols, flavan-3-ols, flavonols, and tannins have received the most attention due to their higher antimicrobial activity compared to other polyphenols and the fact that most of them are capable of suppressing several virulence factors such as inhibiting biofilm formation and neutralizing bacterial toxins [60, 61].

### 4.4. Principal Component Analysis (PCA)

Principal Component Analysis (PCA) is one of the most widely used descriptive methods for determining correlations between a group of quantitative variables and individuals. The results of the present study revealed that the extracts richer in polyphenols have a better inhibition activity via the alpha-glucosidase test and the inhibition of bacterial growth mainly of the *Pseudomonas aeruginosa* strain; on the other hand, the extracts rich in tannins presented a better correlation with the 6 microbial strains. The interesting properties of the extracts that are rich in polyphenols and tannins with their antioxidant activities have been reported in our previous work that focused on the analysis of the correlation between flavonoids, tannins, and polyphenols and the five methods used for the evaluation of antioxidant activity (DPPH, RP, ABTS, FRAP, and H_2_O_2_), from which we found that the extracts richest in polyphenols (acetone macerated and ethanolic macerated) were those that presented the best free radical scavenging activity [23]. The PCA performed by Senhaji et al. on the aqueous and organic extracts of *Ajuga iva* spp. *Pseudoiva* from the region of Taza studied by our team under the same experimental conditions also showed a better correlation between the ABTS test of antioxidant activity and polyphenols [62]. These results are in agreement with previous studies that reported that phenolic compounds play an important role in scavenging free radicals and therefore may prevent the onset of diabetes disease [5, 43]. Similarly, phenolic compounds have been shown to possess strong antibacterial activity [60]. The better correlation that we obtained of the extracts rich in tannins (diethyl ether macerated and acetone macerated) with the microbial strains *Escherichia coli*, *Proteus mirabilis*, *Staphylococcus aureus*, and *Bacillus subtilis* can be explained by their antibacterial powers that have been known since the 1990s, where it was demonstrated that these compounds present in green tea (*Camellia sinensis*) inhibited the growth *in vitro* of various bacteria, such as *Vibrio cholerae*, *Streptococcus mutans*, *Campilobacter jejuni*, *Clostridium perfringes*, and *Escherichia coli* [60].

## 5. Conclusion

The present study allowed us to reveal for the first time the mineral composition and hypoglycemic properties via the three tests of inhibition of alpha-amylase, alpha-glucosidase, and beta-galactosidase enzymes of Moroccan *Juglans regia* bark. In addition, it allowed us to make a comparison of the antibacterial activity of organic extracts of Moroccan *J. regia* bark prepared by different methods and modalities of extractions with other *J. regia* harvested in other countries of the world.

At the end of the study of the mineral composition, we can say that *J. regia* is an important source of mineral elements, with very high contents of Fe (19849.8), K (3487.8), Mg (2631.03), P (691.02), Na (515.75), Ca (256.98), Cu (189.75), and Sr (18.18) mg/kg of plant material.

The results of the evaluation of the antidiabetic activity *in vitro* showed that all the extracts of *J. regia* bark and in particular the two extracts prepared cold, the aqueous and acetone macerates presented the best activity of inhibition of the enzymes alpha-amylase, alpha-glucosidase, and beta-galactosidase. However, the choice of the part of the plant to be tested is a determining factor for pharmacological activity.

For the results of antibacterial activity, the macerated acetone extract at the concentration of 80 mg/mL was found to be the most active by inducing the inhibition zone diameters of 12, 17, 18, 11, 14.5, and 16 mm against *Escherichia coli*, *Proteus mirabilis*, *Pseudomonas aeruginosa*, *Staphylococcus aureus*, *Bacillus subtilis*, and *Listeria innocua*, respectively.

The pharmacological properties of the bark of *J. regia* are due to the richness of this plant in polyphenols, flavonoids, tannins, anthraquinones, sterols, saponins, and quinones in the bark of *J. regia* Moroccan [23].

The Principal Component Analysis (PCA) allowed us to find that the best correlations were obtained between the extracts richest in polyphenols and tannins (macerated acetone and macerated diethyl ether) with the tests of the antidiabetic and antibacterial activity.

The results of the present study justified the use of the bark of *Juglans regia* as an antibacterial and antiseptic agent by the population in the treatment of dental infections.

## Figures and Tables

**Table 1:** Mineral composition of *Juglans regia* L. bark expressed as mg/kg of plant material.

Mineral elements	Ca	Cu	Fe	K	Mg	Na	P	Sr	Se	Zn
Content (mg/kg)	256.98	189.75	**19849.8**	**3487.8**	**2631.03**	515.76	**691.02**	18.18	nr	nr

nr: not reported; the content was under the detection limit.

**Table 2:** Median inhibitory concentrations (IC_50_ in *μ*g/mL) of aqueous and organic extracts of *Juglans regia* L. bark on alpha-amylase, alpha-glucosidase, and beta-galactosidase enzyme inhibition assays.

Extracts from *Juglans regia* bark		Alpha-amylase (IC_50_*μ*g/mL)^x^	Alpha-glucosidase (IC_50_*μ*g/mL)^x^	Beta-galactosidase (IC_50_*μ*g/mL)^x^
Aqueous	Decocted	16161 ± 62.61^a^	463.73 ± 7.36^a^	1406.33 ± 9.66^a^
	Infused	8689.66 ± 318.23^b^	420.8 ± 1.73^b,a,c^	1032 ± 24.91^b^
	*Macerated*	13424.33 ± 284.55^c^	414.46 ± 13.98^c,a^	960.1 ± 38.43^c,b^
Organic	Ethanolic extract	7806.33 ± 85.08^d,b^	488.83 ± 36.34^d,a,b,c^	586.7 ± 25.77^d^
	Macerated ethanolic extract	7595.33 ± 148.6^e,d,b^	497.93 ± 11.93^e,d,a,b,c^	932.26 ± 7.72^e,b,c^
	Acetone extract	13012.33 ± 382.95^f,c^	306.9 ± 1.88^f,c^	919.46 ± 41.28^f,e,b,c^
	*Macerated acetone extract*	5445.33 ± 82.58^g^	323.7 ± 1.71^g,f,b,c^	811.2 ± 8.32^g,e,f^
	Diethyl ether extract	7298.66 ± 46.65^h,d,e^	619.5 ± 10.48^h^	1277 ± 28.59^h,a^
	Macerated diethyl ether extract	7810.33 ± 95.2^i,d,e,b,h^	488.83 ± 36.34^i,d,e,a,b,c^	901 ± 10.91^i,e,f,g,b,c^
Reference standard	Acarbose	616.33 ± 6.58^j^	195 ± 5^j^	—
	Quercetin	—	—	171.16 ± 2.90^j^

(i) Data are expressed as mean ± standard deviation (*n* = 3). (ii) Different letters in the same column indicate a significant difference (*p* < 0.05). (iii) ^x^Concentration that inhibits 50% of the activity in micrograms per milliliter (*μ*g/mL).

**Table 3:** Inhibition zone diameters of different organic extracts from *Juglans regia* L. bark by the disc diffusion method.

Bacterial species/extracts	Ethanolic extract			Macerated ethanolic extract			Acetone extract			Macerated acetone extract			Diethyl ether extract			Macerated diethyl ether extract			Standard (+)		Standard (-)
	20 mg/mL	40 mg/mL	80 mg/mL	20 mg/mL	40 mg/mL	80 mg/mL	20 mg/mL	40 mg/mL	80 mg/mL	20 mg/mL	40 mg/mL	80 mg/mL	20 mg/mL	40 mg/mL	80 mg/mL	20 mg/mL	40 mg/mL	80 mg/mL	Tetra 20 *μ*g/mL	Ak 30 *μ*g/mL	DMSO (10%)
B.G-																					
Ecoli	9	9.5	**11**	8	9	**10**	9	10	**12**	11	12	**12**	7	8	**12**	7.5	9	**14**	12	0	0
Prote	9	11.5	**14**	9	12.5	**14.5**	9	13	**15.5**	11	13	**17**	10	12	**15.5**	13.5	15	**21.5**	23	0	0
Psdm	11	8.5	**14**	9.5	11	**14.5**	11	11.5	**14.5**	12	15.5	**18**	0	7	**7**	0	7	**10**	0	21	0
B.G+																					
Staph	0	0	**10**	0	0	**10**	7.5	10	**11**	10	10	**11**	7	11	**11**	11.5	12.5	**14.5**	13	0	0
Bacil	9	10.5	**11**	9	9	**10**	10	11.5	**12.5**	10	12	**14.5**	11	12.5	**15.5**	14	15	**20**	0	24.5	0
Lister	11	11	**15**	9	10.5	**12.5**	9.5	11.5	**11.5**	11	15	**16**	7	8.5	**9.5**	0	9	**11**	0	26.5	0

B.G-: Gram-negative bacteria. Ecoli: *Escherichia coli* K12. Prote: *Proteus mirabilis*. Psdm: *Pseudomonas aeruginosa* CECT118. B.G+: Gram-positive bacteria. Staph: *Staphylococcus aureus* CECT976. Bacil: *Bacillus subtilis* DSM6633. Lister: *Listeria innocua* CECT 4030. Tetra: tetracycline. Ak: amikacin. DMSO: dimethyl sulfoxyde.

**Table 4:** Minimal inhibitory concentration and minimal bactericidal concentration (MIC and MBC/mg/mL) of different organic extracts of *Juglans regia* L. bark.

Bacterial species/extracts	Ethanolic extract			Macerated ethanolic extract			Acetone extract			Macerated acetone extract			Diethyl ether extract			Macerated diethyl ether extract		
	MIC	MBC	R	MIC	MBC	R	MIC	MBC	R	MIC	MBC	R	MIC	MBC	R	MIC	MBC	R
B.G-																		
Ecoli	**2.5**	80	32	**2.5**	Nd	Nd	**1.75**	Nd	Nd	**1.75**	80	46	**5**	Nd	Nd	**5**	Nd	Nd
Prote	**0.87**	Nd	Nd	**0.87**	Nd	Nd	**1.75**	Nd	Nd	**0.87**	Nd	Nd	**1.75**	Nd	Nd	**1.75**	Nd	Nd
Psdm	**0.87**	40	46	**1.75**	40	23	**1.75**	80	23	**0.87**	40	46	**5**	80	16	**5**	80	16
B.G+																		
Staph	**2.5**	Nd	Nd	**2.5**	Nd	Nd	**2.5**	Nd	Nd	**1.75**	80	46	**5**	Nd	Nd	**5**	Nd	Nd
Bacil	**5**	Nd	Nd	**2.5**	Nd	Nd	**2.5**	Nd	Nd	**2.5**	Nd	Nd	**5**	Nd	Nd	**5**	Nd	Nd
Lister	**0.87**	40	46	**0.87**	40	46	**1.75**	80	46	**0.87**	20	23	**1.75**	80	46	**1.75**	80	46

B.G-: Gram-negative bacteria. Ecoli: *Escherichia coli* K12. Prote: *Proteus mirabilis*. Psdm: *Pseudomonas aeruginosa* CECT118. B.G+: Gram-positive bacteria. Staph: *Staphylococcus aureus* CECT976. Bacil: *Bacillus subtilis* DSM6633. Lister: *Listeria innocua* CECT 4030. MIC: minimal inhibitory concentration. MBC: minimal bactericidal concentration. R: ratio MBC/MIC. Nd: not determined.

**Table 5:** Correlation matrix between the results of polyphenols, flavonoids, tannins, alpha-amylase, alpha-glucosidase, beta-galactosidase, and the results of antibacterial activity by the disc diffusion method.

Variables	Polyphenols	Flavonoids	Tannins	Alpha-amylase	Alpha-glucosidase	Beta-galactosidase	Ecoli	Prote	Psdm	Staph	Bacil	Lister
Polyphenols	**1**	-0.1340	-0.5719	-0.0811	**0.7904**	0.4654	-0.4737	-0.3352	**0.9595**	-0.4564	-0.6046	0.6831
Flavonoids		**1**	0.3999	0.3819	0.0787	-0.6387	0.5037	0.6569	-0.1307	0.5585	0.6466	-0.2714
Tannins			**1**	-0.1690	0.0359	-0.3230	**0.9511**	**0.7320**	-0.5145	**0.7969**	**0.8818**	-0.4615
Alpha-amylase				**1**	-0.2583	0.0709	-0.0349	0.1449	0.1233	-0.0064	0.1824	0.4520
Alpha-glucosidase					**1**	0.2996	0.0769	0.0314	**0.7769**	-0.0598	-0.1447	0.4799
Beta-galactosidase						**1**	-0.2031	-0.1635	0.6076	-0.2044	-0.3470	**0.8019**
Ecoli							**1**	**0.8994**	-0.3872	**0.9259**	**0.9512**	-0.3289
Prote								**1**	-0.2543	**0.9776**	**0.9220**	-0.2338
Psdm									**1**	-0.4017	-0.5076	**0.8515**
Staph										**1**	0.9315	-0.3815
Bacil											**1**	-0.3942
Lister												**1**

Ecoli: *Escherichia coli* K12. Prote: *Proteus mirabilis*. Psdm: *Pseudomonas aeruginosa* CECT118. Staph: *Staphylococcus aureus* CECT976. Bacil: *Bacillus subtilis* DSM6633. Lister: *Listeria innocua* CECT 4030.

## Data Availability

The experimental data used to support the findings of this study are incorporated into the article.

## Abbreviations

*J. regia*: *Juglans regia* L.
DMSO: Dimethyl sulfoxide
MIC: Minimum inhibitory concentration
MBC: Minimum bactericidal concentration
PCA: Principal Component Analysis
ANOVA: Analysis of variance
IC_50_: Concentration that causes 50% inhibition
CFU: Colony-forming unit
WHO: World Health Organization
mm: Millimeter
h: Hours.
